# Aberrant Partial Chromosomal Instability With Chemotherapeutically Resistant Metachronous Colorectal Cancer Following a Synchronous Primary Colorectal Cancer: A Case Report

**DOI:** 10.7759/cureus.11308

**Published:** 2020-11-03

**Authors:** Jerry Lorren Dominic, Shah Huzaifa Feroz, Abilash Muralidharan, Asma Ahmed, Pragatheeshwar Thirunavukarasu

**Affiliations:** 1 General Surgery, South Texas Health System, McAllen, USA; 2 General Surgery, Jawaharlal Nehru Medical College, Aligarh, IND; 3 General Surgery, Larkin Community Hospital, Miami, USA; 4 Internal Medicine, Kiruba Hospital, Coimbatore, IND; 5 General Surgery, Ramaiah Medical College and Hospital, Bangalore, IND; 6 Surgical Oncology, Cape Fear Valley Medical Center, Fayetteville, USA

**Keywords:** primary colorectal cancer, metachronous colorectal cancer, synchronous colorectal cancer, chromosomal instability, microsatellite instability, metastatic colorectal cancer, chemotherapeutic resistance, capecitabine, cetuximab, irinotecan

## Abstract

The diagnosis of synchronous colorectal cancer (CRC) is crucial as the management, including the extent of surgical resection, depends on it. There have been numerous studies on the clinicopathological features of synchronous CRC; however, only a few studies have discussed synchronous cancer treatment. The guidelines to best manage the synchronous and metachronous CRC are limited, especially the most appropriate surgical treatment and chemotherapy based on mutational analysis of mismatch repair genes and the carcinoma sequence model. We present a rare case of a metachronous CRC with intact nuclear expression of microsatellite instability markers following a synchronous CRC, and it failed to show any significant response to surgical resection and chemoradiotherapy.

A 53-year-old female presented in June 2016 with bleeding per rectum for one month, weight loss, and a recent history of altered bowel habits. The per rectal examination revealed a circumferential growth. Colonoscopy and biopsy yielded multiple polyps throughout the colon and invasive adenocarcinoma in the upper and lower one-third of the rectum. The above features were highly suggestive of synchronous CRC. Serologic studies revealed elevated carcinoembryonic antigen (CEA). Excisional biopsy of mesenteric and retroperitoneal lymph nodes during proctocolectomy and end ileostomy was negative for metastasis, including the other metastatic workup preoperatively-eight months post-resection and adjuvant chemotherapy patient developed metachronous CRC. Mutational analysis showed positivity only for *adenomatous polyposis coli *(APC) while negative for *KRAS, NRAS*, and *BRAF*. Immunohistochemistry (IHC) markers for mismatch repair (MMR) proteins showed intact protein expression. The patient was given multiple chemotherapy cycles throughout her course, including oral capecitabine, XELOX (capecitabine + oxaliplatin), cetuximab-capecitabine, cetuximab-irinotecan, and FOLFIRI (5‐fluorouracil [5‐FU] + irinotecan + folinic acid)-bevacizumab, as is the standard chemotherapy regimen for these tumors.

The diagnosis of metachronous CRC with intensive follow up is crucial. IHC markers for MMR proteins showed intact protein expression ruling out the possibility of microsatellite instability and Lynch Syndrome. The only presence of APC mutation indicates a partial chromosomal instability. During the course, the patient had either stable size of the masses or developed new metastatic growth despite intensive chemotherapeutic regimes. Unfortunately, there are no precise guidelines based on aberrant mutational analysis regarding synchronous and metachronous CRCs management.

## Introduction

Synchronous colorectal carcinoma (synchronous CRC) is a rare type of colorectal malignancy, defined by the presence of more than one primary colorectal carcinoma at the time of initial presentation [1]. The prevalence of all colorectal cancers ranges from 1.1% to 8.1% [2]. The diagnosis of the synchronous CRC is vital. If disregarded, they can develop into advanced-stage metachronous cancer and usually require re-operation with intensive chemotherapy. A preoperative diagnosis of synchronous colorectal cancer is crucial because it may influence the treatment options concerning the type and extent of surgical resection [3]. There are numerous studies on the clinicopathological characteristics of synchronous cancer. However, very few studies have reviewed the treatment of synchronous cancer. The guidelines to best manage the synchronous CRC are limited, primarily the most appropriate surgical treatment and chemotherapy based on mutational analysis results of mismatch repair genes and the adenoma-carcinoma sequence model.

Metachronous colorectal cancers are defined as primary colorectal cancers developing six months after the previous colorectal surgery for the CRC [4]. Although the incidence of metachronous CRCs is increasing, the management guidelines, including chemotherapy, prognosis, and the impact of synchronicity on the prognosis, also remain understudied.

Our case report presents a rare metachronous CRC following a synchronous CRC. In addition, it exhibits partial chromosomal instability with intact nuclear expression of microsatellite instability markers. Moreover, the CRC failed to show any significant response to surgical resection concomitant with chemoradiotherapy.

## Case presentation

A 53-year-old female presented in June 2016 with bleeding per rectum for one month, 10-pound weight loss over nine months, and a recent history of altered bowel habits. The patient denied nausea, vomiting, abdominal discomfort, fevers, bone pain, night sweats, trauma, intake of blood thinners. Her past medical history and family history were non-significant. On physical examination, the patient was obese. Her vitals were within normal limits, and exhibited mild pallor. The abdomen was soft, obese, non-distended with a mild left lower quadrant tenderness with no abnormal mass in any quadrant or any clinical evidence of cirrhosis. On per rectal examination, the circumferential growth was evident.

This constellation of symptoms necessitated an extensive workup. The patient underwent colonoscopy and biopsy, which revealed a rectal growth to be grade II-III moderately differentiated adenocarcinoma. In addition, multiple adenomatous polyps with low-grade dysplasia extending from the cecum to descending colon were present.

CT scan of the abdomen revealed multiple colonic polyps throughout the colon and circumferential mass/large polyps in the distal sigmoid colon, rectosigmoid junction, and rectum extending for a length 9-10 cm. The liver was enlarged and showed diffuse-low attenuation consistent with the fatty infiltration with no focal nodule or area of enhancement. No significant lymphadenopathy was apparent. Other workups for metastasis were either negative or normal.

Serum lab profile (including CBC, CMP, coagulation studies, LFT, RFT) was positive only for mild anemia (Hb 11.1). The remainder were negative or normal. The serum tumor marker carcinoembryonic antigen (CEA) was elevated 316.9 ng/mL.

A probable initial diagnosis of familial adenomatous polyposis degenerating into synchronous primary colorectal cancer was made. The surgical team was consulted for further evaluation and management.

In view of the low rectal cancer, the patient underwent total proctocolectomy with end ileostomy with adjacent lymph node resection in June 2016. The gross specimen revealed more than 200 mostly sessile polyps throughout the colon with an ulcero-proliferated lesion studded in the upper third of the rectum and the largest polyp in the lower third of the rectum. Morphologic review of the slides from ulcero-proliferated lesion demonstrated high-grade/poorly differentiated malignant neoplasm with pleomorphic hyperchromatic nuclei infiltrating the muscularis propria. At the same time, the section from the largest polyp revealed invasive adenocarcinoma infiltrating the submucosa. The histopathology of the resected 29 lymph nodes (22 mesocolic, 7 inferior mesenteric) was negative for any malignancy.

Staging (2010 AJCC TNM Classification System for Colorectal Cancer [5]): pT2: Tumor invading the muscularis propria in a sample from the distal ulcero-proliferated lesion (12 cm from the distal resection margin) pT1: Tumor invading submucosa in a sample from the largest polyp (2 cm from the distal resection margin) pN0: No regional lymph node metastasis M0: No distant metastasis by imaging; no evidence of tumor in other sites or organs

Given the above parameters, an initial diagnosis of stage I primary colorectal cancer (CRC) was made.

In light of the familial polyposis nature and early colorectal cancer, the patient received adjuvant chemotherapy from July 2016 to September 2016 with oral capecitabine (two cycles of 21 days). Concurrently, she received the 28 fractions of 50.4 cGY radiotherapy.

**Figure 1:**
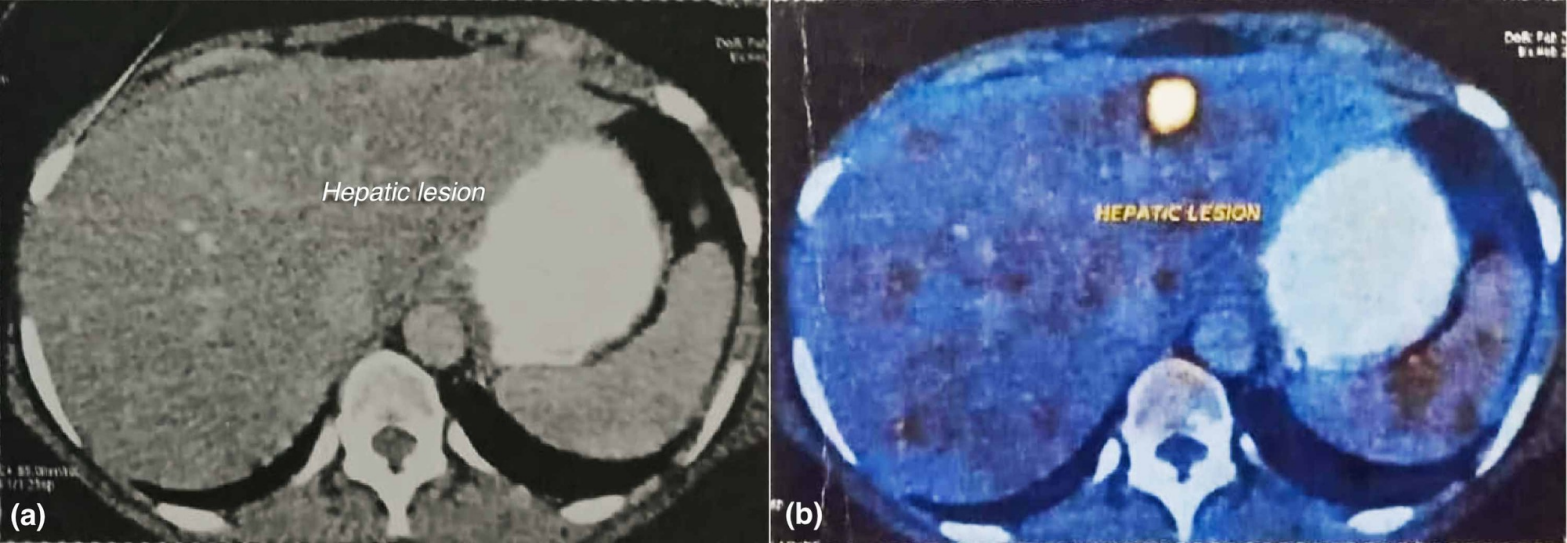
(a) Contrast-enhanced CT abdomen image shows solitary hepatic metastasis. (b) Corresponding fused PET-CT image shows a metabolically active hypodense lesion of size 16 x 14 mm in segment II/III of the left lobe of the liver (standardized uptake value [SUV] Max = 5.5). PET-CT, positron emission tomography-computed tomography.

**Figure 2:**
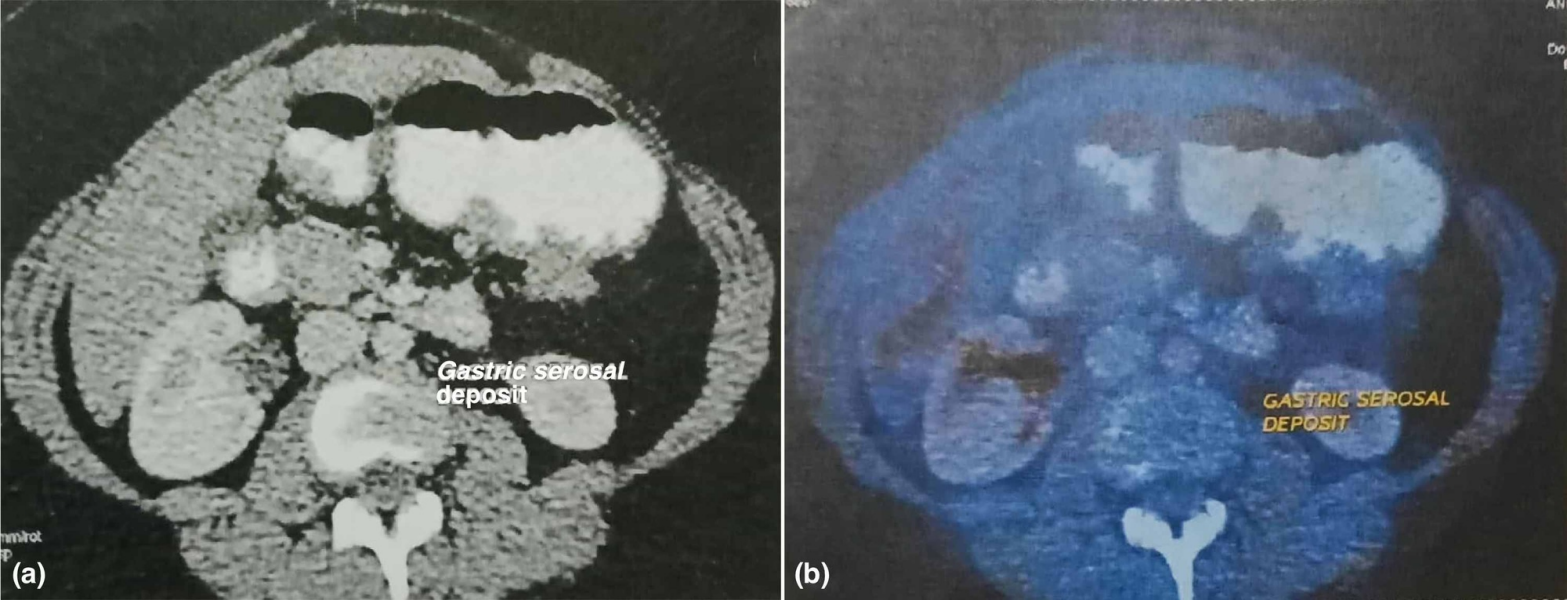
(a) Contrast-enhanced CT abdomen image shows gastric serosal deposits. (b) Corresponding fused PET-CT image shows gastric serosal deposits. A peritoneal nodule of size 25 x 20 mm is noted abutting the greater curvature of the stomach with mild metabolic activity. PET-CT, positron emission tomography-computed tomography.

**Figure 3:**
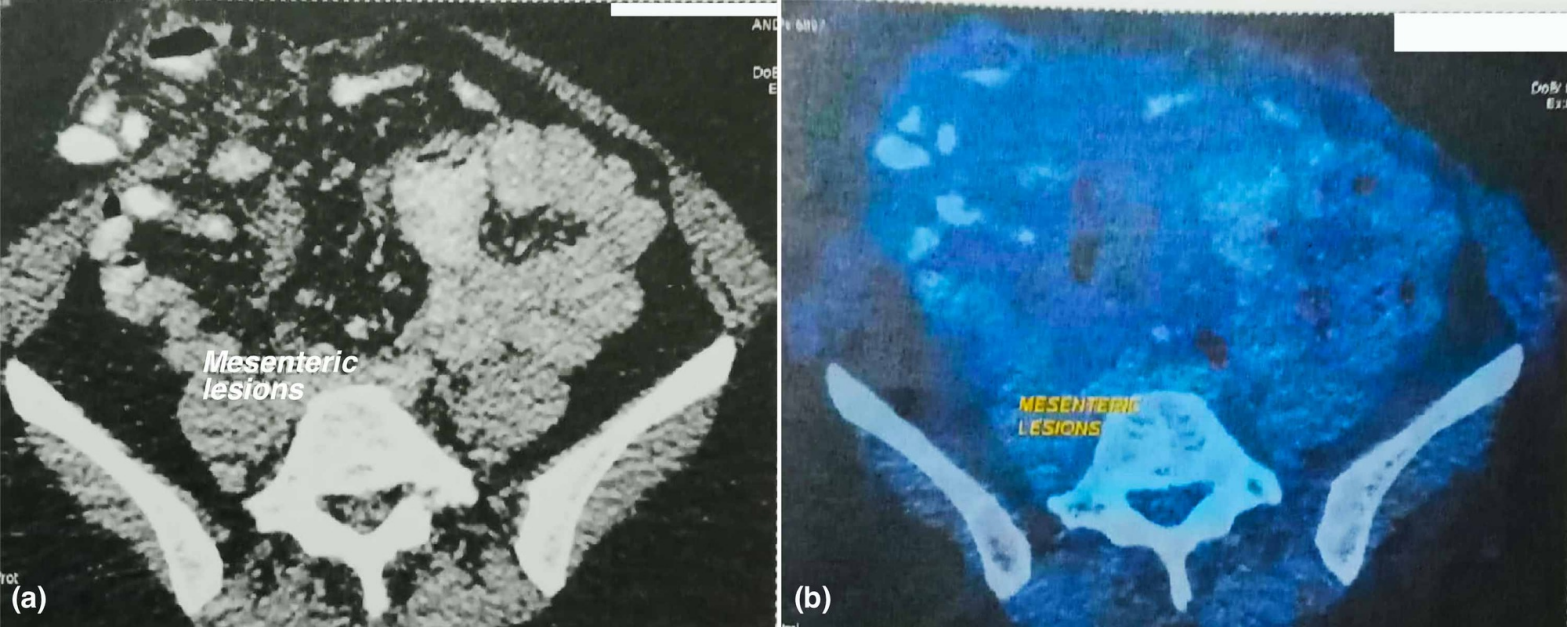
(a) Contrast-enhanced CT abdomen image shows mesenteric lesions with peritoneal fat stranding. (b) Corresponding fused PET-CT image shows patchy areas of soft tissue thickening in the mesentery adjacent to the ileostomy site with mild increased metabolic activity, measuring ~ 28 x 18 mm (standardized uptake value [SUV] Max = 1.5). PET-CT, positron emission tomography-computed tomography.

Positron emission tomography-computed tomography (PET-CT) was subsequently obtained in February 2017, which revealed extensive FDG-avid hepatic, peritoneal and mesenteric metastases (Figures 1, 2, 3).

Furthermore, the genetic analysis of the specimen acquired from the liver biopsy showed strong positivity for *APC* while negative for *BRAF*, *KRAS*, and *NRAS* (Table 1).

**Table 1:** PCR amplified DNA sequencing for mutation analysis of the specimen from liver metastasis

PCR Amplified DNA Sequencing for Mutation Analysis	Result(s)
APC	Positive
BRAF	Negative
KRAS	
NRAS	

In light of advanced metastatic disease, six cycles of XELOX regimen chemotherapy was planned in a three-week treatment cycle: IV oxaliplatin 130 mg/m² (day 1) followed by PO capecitabine 1000 mg/m² twice daily (day 1 evening to day 15 morning) with no treatment for seven days. After completion of the five cycles of chemotherapy, a repeat PET-CT was obtained in June 2017 to evaluate his response to treatment. It yielded a resolution of hepatic metastasis in the left lobe; however, the peritoneal and mesenteric deposits remained stable and non-FDG avid. The irregular peritoneal deposits were seen indenting the stomach's posterior body along the greater curvature and measured 2.2 x 1.4 cm (previously 2.5 x 2.0 cm), while the mesenteric deposits measure 2.7 x 1.7 cm (previously 2.8 x1.8 cm) in the largest dimension. During cycle 6 (May 2017), the patient's treatment course was complicated by chemotherapy-induced severe neutropenia on CBC, with no additional symptoms.

In July 2017, given the mild response and advanced metastatic disease, the patient was further advised 12 cycles of combination chemotherapy regimen (cetuximab-capecitabine) in a two-week cycle (weekly cetuximab (400 mg/m² [over 120 minutes], followed by 250 mg/m² [over 30 minutes]), capecitabine 800 mg/m² twice daily (day 1 through 14, every two weeks)).

**Figure 4:**
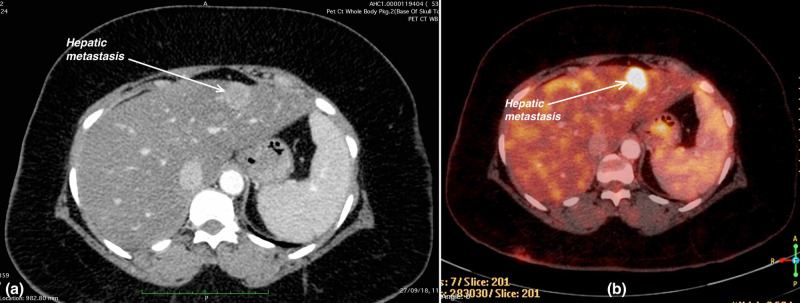
(a) Contrast-enhanced CT abdomen image shows a new solitary hepatic metastasis in the left lobe of the liver. (b) Corresponding fused PET-CT image shows a new metabolically active hypodense lesion of size 20 x 16 mm in the left lobe of the liver. PET-CT, positron emission tomography-computed tomography.

Following the completion in Jan 2018, the patient was given an additional eight-week cycle of cetuximab-irinotecan (weekly cetuximab (400 mg/m² [over 120 minutes], followed by 250 mg/m² [over 30 minutes]), IV Irinotecan 200 mg/m² [on day 1 every 2 weeks]) until August 2019. PET-CT obtained in September 2018 revealed new hypermetabolic solitary hepatic metastasis (Figure 4) along with new non-FDG avid parenchymal nodules in the upper lobe of the right lung.

A new combination of chemotherapy FOLFIRI and Bevacizumab in a 2 weekly cycle was started in September 2019, considering the new metastatic lesions despite being on cetuximab-irinotecan chemotherapy: Day 1: IV irinotecan 180 mg/m² [over 30-90 minutes] with leucovorin 400 mg/m² IV infusion to match the duration of irinotecan infusion, followed by: Days 1-2: fluorouracil 400 mg/m² IV push on day 1, then 1,200 mg/m²/day × 2 days (total 2,400mg/m² over 46-48 hours) IV continuous infusion Day 1: bevacizumab 5mg/kg IV

**Figure 5:**
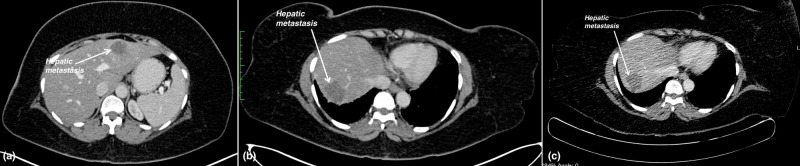
(a) PET-CT shows hepatic metastasis in the left lobe of the liver. (b) PET-CT shows hepatic metastasis in the right lobe of the liver. (c) PET-CT shows stable hepatic metastasis in the right lobe of the liver. PET-CT, positron emission tomography-computed tomography.

PET-CT obtained sequentially revealed: October 2019: Hypermetabolic metastatic lesions in both lobes of the liver (Figure 5a, b) with mildly FDG avid metastatic nodules in the upper lobe of the right lung. January 2020: Stable size (Figure 5c) and activity September 2020: Multiple metastatic lesions in various segments of the liver.

Considering she was non-responsive to chemotherapy, the surgical team, as per the National Comprehensive Cancer Network (NCCN) guidelines version 4.2020, planned to perform a needle core biopsy from the metastatic lesion in the left lobe of the liver for immunohistochemistry testing, to detect microsatellite instability or mismatch repair, to guide chemotherapeutic regime. The results were typical for each marker (i.e., all four MMR proteins are normally expressed). Additionally, Her-2 testing is also negative (Table 2).

**Table 2:** Immunohistochemistry (IHC) studies for mismatch repair (MMR) proteins and Her-2/Neu protein.

Immunohistochemistry (IHC) Markers	Pattern of Expression
MLH1	Intact protein expression
MSH2	Intact protein expression
MSH6	Intact protein expression
PMS2	Intact protein expression
Her-2/Neu	Negative

## Discussion

CRC is the most common gastrointestinal cancer and is the third most common malignancy in the United States and the third leading cause of cancer-related deaths. CRC has both strong environmental associations and genetic risk factors. The incidence rate peaks in the 7th-8th decades (but increases with age starting at 40), with equal incidence in males and females. The incidence and mortality rate has been steadily declining for the past years, due to increased cancer screening and better therapy modalities.

**Figure 6:**
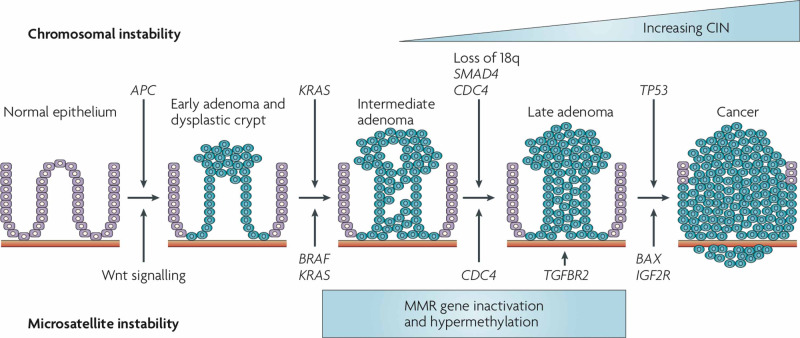
Adenoma–carcinoma sequence model for microsatellite and chromosomal instability in colorectal cancer. This model is likely to be an oversimplification. Still, it represents the clinicopathological changes with genetic abnormalities in the progression of chromosomally unstable colorectal cancer (CRC) (the gatekeeper pathway involving genes that regulate cell growth). From [7].

CRC development is a multistep process wherein benign adenomas give rise to carcinomas (Figure 6) [6]. Sequential cellular and molecular events in the mucosal epithelium lead to altered proliferation, cellular accumulation, and glandu­lar disarray, which may result in adenomatous polyps. Further genetic alteration results in a greater degree of cellular atypia and glandular disorgani­zation (dysplasia), which may evolve into a carcinoma. The sequence of adenoma-to-carcinoma is almost always associated with genetic changes, even in sporadic colon cancers. Multiple somatic mutations contributed by environmental insults are associated with polyps and cancers. The genetic contribution in the formation of adenomas, as well as progression to malignancy, include: Mutation in *proto-oncogenes (K-ras)* Inactivation of tumor suppressor gene: Stepwise progression of the tumor is associated with inactivation of tumor suppressor gene designated *DCC (deleted in colorectal cancer) *on chromosome 18q- (responsible for normal cell-cell adhesive interactions) in more than 75% of cases, i.e., 18q deletion is the most common cytogenetic abnormality. While chromosome 17p deletions can cause loss of the *p-53 tumor suppressor gene*. Mutation in genes involved in DNA repair (mismatch repair genes) can cause microsatellite instability (a clinically significant prognostic factor recommended in TNM 2010 staging criteria).

Synchronous CRC, as suggested in the previous studies, are more common in the setting of diseases with a precursor lesion such as adenoma as in hereditary nonpolyposis colorectal cancer (HNPCC; Lynch Syndrome), familial adenomatous polyposis, and inflammatory bowel diseases [2]. Typical sites of synchronous CRC are the rectum and sigmoid colon. The lack of *KRAS* and *BRAF* mutation and no loss of nuclear expression of mismatch repair proteins (MLH1, MSH2, MSH6, PMS2) indicates a low probability of microsatellite instability. It also ruled out the presence of Lynch syndrome. In contrast to our case, the synchronous CRC is more heeded in the colon's proximal portion, has a higher incidence of mucinous adenocarcinoma, and a higher likelihood of having microsatellite instability [2].

The diagnosis of synchronous CRC has increased, primarily because of advancements in diagnostic modalities such as colonoscopy and computed tomography (CT) colonography [8]. However, there is much skepticism about the most appropriate surgical treatment. Few authors have suggested that the treatment of synchronous or metachronous CRC is the same as solitary colorectal carcinoma, with the removal of enough bowel and local lymph nodes resection [9]. It is proposed that subtotal colectomy with recto-ileostomy should be performed for patients with distant synchronous lesions, combined with multiple adenomatous polyps or a familial history if they are a surgical candidate [9]. Others suggest extensive surgical resection is required for patients with synchronous colorectal cancer with known predisposing factors such as familial adenomatous polyposis, HNPCC or ulcerative colitis [2]. For other cases, appropriate surgical resection with a colonoscopic examination on follow-up is recommended. Passman et al. [10] suggested a more extensive resection for lesions in adjacent segments. Some authors have recommended multiple resections aimed at retaining the normal colon [11,12]. Likewise, there has been insufficient agreement among surgeons regarding the appropriate surgical treatment for synchronous cancers located in separate segments.

The patient manifested early metachronous metastases as the metastatic lesions were detected within 12 months after the surgery of the primary CRC. The current National Comprehensive Cancer Network (NCCN) guidelines for unresectable metastatic CRC recommend FOLFIRI (5‐fluorouracil [5‐FU] + irinotecan + folinic acid) or FOLFOX (5‐FU + oxaliplatin + folinic acid) or CAPEOX/XELOX (capecitabine + oxaliplatin), or FOLFOXIRI (5‐FU + oxaliplatin + irinotecan + folinic acid) ± bevacizumab for patients who are appropriate for intensive therapy [13]. The NCCN panel recommends these regimens as uniformly effective cytotoxic treatment options. Little or no clinical difference is discerned when administering intensive therapy as first‐line versus subsequent‐line therapy following less intensive therapy [13-17]. The European Society for Medical Oncology guidelines similarly recommends first‐line backbone chemotherapy of a fluoropyrimidine in various schedules and combinations [18]. FOLFOX and FOLFIRI are currently considered the preferred cytotoxic treatment options for first‐line treatment of mCRC [14, 18].

The chemotherapy and immunotherapy based on mutational analysis of the specimen for the metachronous metastatic CRC remain understudied. For example, it is clearly understood the absence of KRAS mutation makes the EGFR specific antibody ineffective. So the question arises what chemotherapy or immunotherapy or their combination would be most effective if the patient shows positivity for *APC* genes while negative for *KRAS*, *NRAS*, and *BRAF* on DNA sequencing, i.e., a patient showing a partial chromosomal instability as per the adenoma-carcinoma sequence model. Concurrently, there are no established guidelines for optimal sequencing of biological or cytotoxic agents in metastatic colorectal cancer (mCRC) [19].

The prognosis of synchronous CRC compared to solitary CRC also remained dubious. The retrospective analysis performed by Passman et al. [10] showed a similar prognosis between the two. While in multivariate analysis, synchronous CRC was an independent prognostic factor associated with poor overall survival [1,20]. As yet, no biological marker has been recognized that distinguishes synchronous metastases from metachronous metastases [20].

## Conclusions

Classifying patients at high risk for developing metachronous colorectal cancer is crucial as it may contribute to more accurate patient information, customized follow-up plans, and adequate management. The chemotherapy proposed for the metachronous CRC also remains widely understudied. Unfortunately, there are no precise guidelines based on mutational analysis regarding the management of metachronous CRC post resection of the synchronous CRC. As such, the selection of a chemotherapeutic agent is based on less studied trials and depends on the patient’s clinical status and the clinician’s expertise. Until now, no data are present in the literature regarding the management of metachronous colorectal malignancy, which showed the partial mutational characteristics of chromosomal instability. This patient’s aberrant mutational analysis in the presence of his elevated serum tumor markers and chemotherapeutic resistance indicates a need for more specific modalities of diagnosis, treatment, and follow-up.
